# *Linum usitatissimum AccD* Enhances Seed Fatty Acid Accumulation and Tolerance to Environmental Stresses during Seed Germination in *Arabidopsis thaliana*

**DOI:** 10.3390/plants12173100

**Published:** 2023-08-29

**Authors:** Rui Du, Xinye Li, Huan Hu, Yu Zhao, Mingxun Chen, Zijin Liu

**Affiliations:** National Yangling Agricultural Biotechnology & Breeding Center, Shaanxi Key Laboratory of Crop Heterosis and College of Agronomy, Northwest A&F University, Yangling 712100, China; durui000@nwafu.edu.cn (R.D.); lixinye0324@163.com (X.L.); huhuan@nwafu.edu.cn (H.H.); nwafu_zy@163.com (Y.Z.); cmx786@nwafu.edu.cn (M.C.)

**Keywords:** *LuAccD*, fatty acids, salt stress, mannitol stress, seed germination

## Abstract

Flax (*Linum usitatissimum* L.), as an important oil-producing crop, is widely distributed throughout the world, and its seeds are rich in polyunsaturated fatty acids (FAs). Previous studies have revealed that *Arabidopsis thaliana* ACETYL-CoA CARBOXYLASE (AtACCase) is vital for FA biosynthesis. However, the functions of *L. usitatissimum AccD* (*LuAccD*) on FA accumulation and seed germination remain unclear. In the present study, we cloned the LuAccD coding sequence from the flax cultivar ‘Longya 10’, identified conserved protein domains, and performed a phylogenetic analysis to elucidate its relationship with homologs from a range of plant species. Ectopic expression of *LuAccD* in *A. thaliana* wild-type background enhanced seed FA accumulation without altering seed morphological characteristics, including seed size, 1000-seed weight, and seed coat color. Consistently, the expression of key genes involved in FA biosynthesis was greatly up-regulated in the developing seeds of *LuAccD* overexpression lines. Additionally, we demonstrated that LuAccD acts as a positive regulator of salt and mannitol tolerance during seed germination in *A. thaliana*. These results provide important insights into the functions of *LuAccD*, which facilitates the oil quantity and abiotic stress tolerance of oil-producing crops through genetic manipulation.

## 1. Introduction

Flax (*Linum usitatissimum* L., 2n = 30) is a versatile annual plant with global production areas of approximately 12 million acres mainly in Kazakhstan, Russia, Canada, and China; primarily cultivated for its seed oil (oilseed flax) and stem fiber (fiber flax) [1]. Oilseed flax generally contains approximately 50% oil which is composed of five major fatty acids (FAs): palmitic acid (C16:0), stearic acid (C18:0), oleic acid (C18:1), linoleic acid (C18:2), and linolenic acid (C18:3) [2,3]. Among them, the percentage of C18:3 in the flaxseed oil ranges from 40% to 60%, which is significantly higher than that of *Zea mays* (~1%), *Glycine max* (~8%), and *Brassica napus* (~11%) [3]. As polyunsaturated FAs, C18:2 and C18:3 cannot be biosynthesized in the human body and are the precursors for long-chain polyunsaturated FAs, inclusive of arachidonic acid and eicosapentaenoic acid. These long-chain polyunsaturated FAs have a significant role in the prevention of a variety of diseases, including cancers, inflammatory, cardiovascular, and autoimmune diseases [4,5,6]. Therefore, together with a high amount of proteins (up to 18.29%), fiber (27.3%), vitamin B1, and lignans, particularly secoisolariciresinol diglucoside (294–700 mg/100 g) [7,8,9], flax serves as a predominant source which offers a wide range of nutritional and therapeutic applications. In the past decade, China has become the largest importer with the import of $31,108 million, which is equivalent to 26.8% of total global flax import in the year 2020 [10]. However, oilseed flax is mainly grown in the arid and semi-arid regions of the Northern and Northwestern China, which is one of the areas more vulnerable to global climate change [11]. Unpredictable environmental stresses, such as drought and salinity–alkalinity, pose a threat to biological diversity and the quality of oilseed flax. Therefore, identifying the key genes involved in seed FA accumulation and response to adversities in *L. usitatissimum* would provide potential targets for molecular breeding in oil-producing crops including *L. usitatissimum*.

In plants, FA biosynthesis starts with the provision of carbon from glycolysis. After glycolysis, pyruvate dehydrogenase catalyzes the conversion of pyruvate to acetyl-CoA, the initial substrate for de novo FA biosynthesis which occurs in the plastids [12]. Acetyl-CoA carboxylase (ACCase) converts acetyl-CoA and bicarbonate into malonyl-CoA, which is the first committed step in FA biosynthesis [13,14]. In the plastids of dicots and non-graminaceous monocots, ACCase mainly comprises four distinct subunits, namely biotin carboxylase, biotin carboxyl carrier protein, α-subunit of carboxyltransferase (CTα), and β-subunit of carboxyltransferase (CTβ) [15,16]. Studies have shown that increased activity of *A. thaliana* AtACCase in the tuber amyloplasts of *Solanum tuberosum* led to an increase of more than five times in the triacylglycerol content [17]. The mutation of *A. thaliana ACC1* (*AtACC1*), an essential gene encoding ACCase, significantly decreased the contents of long-chain FAs in leaves under cold treatment [18]. Overexpression of *AtACC1* in *B. napus* not only altered seed FA compositions, with the largest effect being an increase in C18:1, but also caused an increase of approximately 5% in seed oil content [19]. Meanwhile, overexpression of each subunit of *Gossypium hirsutum* ACCase effectively increased seed oil content in the transgenic plants of *G. hirsutum*. Among them, the oil content of *GhBCCP1* transgenic seeds was significantly increased by 21.92%, while that of *GhBC1* and *GhCTβ* transgenic seeds was elevated by ~17% [20]. The latest study showed that the interaction between α-CT and CARBOXYLTRANSFERASE INTERACTORs was enhanced by light, which in turn attenuates carbon flux into triacylglycerol accumulation in *A. thaliana* leaves [21]. Homologous expression of *NtAccD*, located in the plastid genome, raised the ACCase level and FA content in the resultant transgenic leaves in *Nicotiana tabacum* cv. *Xanthi* [22]. Semi-quantitative RT-PCR and quantitative real-time PCR (qRT-PCR) results showed that the expression level of *EgAccD* is positively correlated with the *Elaeis guineensis* productivity [23]. The functions of *AccD* genes from *A. thaliana* and other plants have been well characterized, but the roles of *AccD* from *L. usitatissimum* in the regulation of seed FA accumulation and in response to salt and osmotic stresses remain unclear.

For the sessile crops, environmental factors are crucial in determining crop growth and development. Of these, drought and salt are the most prevalent and detrimental constraints to agricultural production [24,25,26,27]. Previous studies have demonstrated that drought can negatively affect the yield potential, oil content and FA compositions, and fiber quality traits of flax [26,28,29]. Meanwhile, soil salinity–alkalinity can result in delayed germination, low seedling survival, irregular growth, and lower yield of flax [27]. In addition, drought can result in osmotic stress by altering water potential and cell turgor, and salt can induce osmotic stress and ion toxicity [30]. It is worth noting that the hyperosmotic signal caused by drought and salt stresses promotes the accumulation of phytohormone abscisic acid (ABA), which in turn triggers a series of adaptive responses in plants [31]. Therefore, ABA biosynthesis and signal transduction are of great importance for plants to resist abiotic stresses.

In this study, we cloned the *LuAccD* gene from the flax cultivar ‘Longya 10′ and found that overexpression of *LuAccD* in *A. thaliana* wild-type plants significantly increased the accumulation of seed total FAs by boosting the transcription levels of several key genes involved in FA biosynthesis. We also demonstrated that LuAccD enhances tolerance to salt and mannitol stresses during seed germination via mediating the ABA biosynthesis and ABA-responsive pathway in *A. thaliana*.

## 2. Results

### 2.1. Sequence Analysis of LuAccD Protein

**Figure 1:**
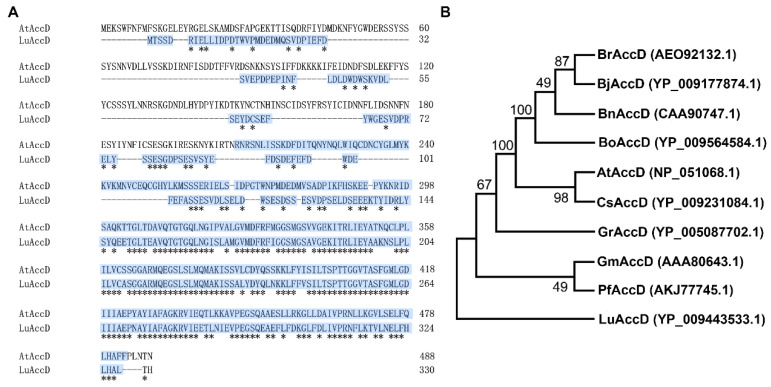
Protein sequence alignment and phylogenetic analysis of AccD. (**A**) Sequence alignment of amino acids from LuAccD and AtAccD. The asterisks represent strictly conserved amino acids. The crotonase-like superfamily domain, which was predicated by the NCBI Conserved Domain Database (https://www.ncbi.nlm.nih.gov/Structure/cdd/wrpsb.cgi, accessed on 20 October 2022), is highlighted in blue in the sequences. (**B**) Phylogenetic analysis of AccD proteins from *L. usitatissimum*, *A. thaliana*, and other crops. Numbers indicate the phylogenetic confidence of the tree topology and denote the bootstrap values on neighbor-joining analysis. Br: *Brassica rapa*, Bj: *Brassica juncea*, Bn: *Brassica napus*, Bo: *Brassica oleracea* var. *oleracea*, At: *Arabidopsis thaliana*, Cs: *Camelina sativa*, Gr: *Gossypium raimondii*, Gm: *Glycine max*, Pf: *Perilla frutescens*, Lu: *Linum usitatissimum*. The accession numbers of AccD are listed in parentheses.

The protein sequence of AtAccD was applied to BLASTP in the Phytozome (https://phytozome-next.jgi.doe.gov/, accessed on 12 October 2020) database and one homologous polypeptide of Lus10002473 was identified from the *L. usitatissimum* genome, namely LuAccD. As shown in Figure 1A, LuAccD and AtAccD had 330 and 488 amino acids, respectively. A 58.4% identity in amino acid sequence was matched between LuAccD and AtAccD, and their carboxyltransferase domains shared 61.5% identity (Table S2). Phylogenetic analysis indicated that LuAccD presents a relatively distant relationship with AccD from other crops we selected (Figure 1B). These results suggested that LuAccD may have a similar function as AtAccD in some ways.

### 2.2. LuAccD Increases the Seed FA Accumulation in A. thaliana

**Figure 2:**
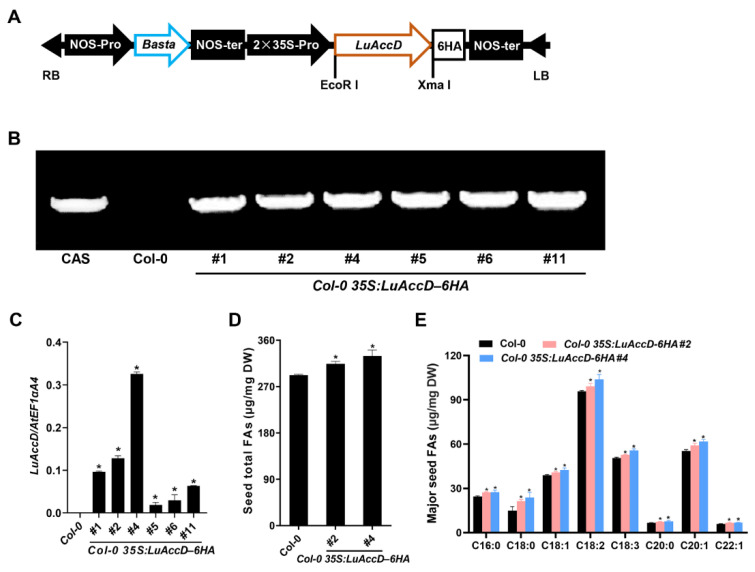
Overexpression of *LuAccD* increased the accumulation of seed FAs in *A. thaliana*. (**A**) Schematic illustration of the constitutive expression cassette of *LuAccD*. RB, right border; LB, left border; NOS-pro, nopaline synthase promoter; NOS-ter, nopaline synthase terminator; Basta, glyphosate; 35S-pro, CaMV 35S promoter. (**B**) PCR-based DNA genotyping of *Col-0 35S: LuAccD–6HA* transgenic plants. Cas, cassette. (**C**) Transcript levels of *LuAccD* in the wild-type (Col-0) and *Col-0 35S: LuAccD–6HA* developing seeds at 12 days after pollination measured by qRT-PCR. *AtEF1αA4* was used as an internal control. The values are presented as the mean ± SD (n = 3). (**D**) Comparisons of seed total FA content between Col-0 and *Col-0 35S: LuAccD–6HA* transgenic plants. (**E**) Comparison of the major seed FA compositions between the Col-0 and *Col-0 35S: LuAccD–6HA* transgenic plants. Values represent means ± SD and error bars denote SD. Three independent experiments were carried out and each biological replicate contains three technical replicates. Asterisks (*) indicate significant differences in the FA contents between *Col-0 35S: LuAccD–6HA* and Col-0 plants (two-tailed paired Student’s *t*-test, *p* ≤ 0.05).

Studies have revealed that the loss of *AtAccD* function results in embryo lethality of *A. thaliana* [32]. To preliminarily investigate the functions of *LuAccD* on the accumulation of seed FAs, we introduced the overexpression construct of *35S: LuAccD–6HA* (Figure 2A) into the *A. thaliana* wild-type (Col-0) plants. We obtained six independent T_3_ homozygous *Col-0 35S: LuAccD–6HA* transgenic lines (#1, #2, #4, #5, #6, and #11) and identified them by the analysis of PCR-based DNA genotyping (Figure 2B). Meanwhile, qRT-PCR results showed that the *LuAccD* expression is not detected in the Col-0, but highly present in the six transgenic lines (Figure 2C). Therefore, we selected *Col-0 35S: LuAccD–6HA#2* and *Col-0 35S: LuAccD–6HA#4* for follow-up experiments. The phenotype analysis showed that there are no significant differences in the seed coat color, seed size, and 1000-seed weight between Col-0 and *Col-0 35S: LuAccD–6HA* transgenic plants (#2 and #4) (Figure S1). However, the contents of seed total FAs and all major FA compositions were both significantly elevated in *Col-0 35S: LuAccD–6HA* plants compared to those in Col-0 (Figure 2D,E). These results suggested that ectopic expression of *LuAccD* promotes FA accumulation without affecting other measured agronomic traits in *A. thaliana* seeds.

### 2.3. LuAccD Increases the Expression Levels of Genes Contributing to Seed FA Accumulation

**Figure 3:**
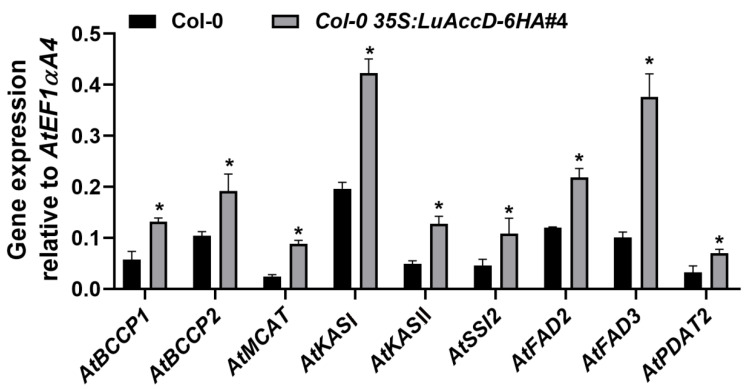
Expression analysis of genes contributing to FA accumulation in the wild-type (Col-0) and *Col-0 35S: LuAccD–6HA#4* developing seeds at 12 days after pollination. Results were normalized against the expression of *AtEF1αA4* as an internal control. Values are means ± SD (n = 3). Asterisks (*) represent significant differences between Col-0 and *Col-0 35S: LuAccD–6HA#4* transgenic plants determined by two-tailed paired Student’s *t*-test (*p* ≤ 0.05).

To further investigate how LuAccD controls seed FA accumulation at transcription level, several key genes inclusive of *AtBCCP1* (*BIOTIN CARBOXYL CARRIER PROTEIN ISOFORM1*), *AtBCCP2*, *AtMCAT* (*MALONYL COA-ACP MALONYLTRANSFERASE*), *AtKASI* (*3-KETOACYL-ACYL CARRIER PROTEIN SYNTHASE I*), *AtKASII*, *AtSSI2* (*SUPPRESSOR OF SA INSENSITIVE 2*), *AtFAD2* (*FATTY ACID DESATURASE2*), *AtFAD3*, and *AtPDAT2* (*PHOSPHOLIPID: DIACYLGLYCEROL ACYLTRANSFERASE2*), were selected for expression analysis. The expression levels of these genes were assessed by qRT-PCR using the developing seeds at 12 days after pollination (DAP) between Col-0 and *Col-0 35S: LuAccD–6HA#4* transgenic plants. The transcript levels of *AtBCCP1*, *AtBCCP2*, *AtMCAT*, *AtKASI*, *AtKASII*, *AtSSI2*, *AtFAD2*, *AtFAD3*, and *AtPDAT2* in the developing seeds of *Col-0 35S: LuAccD–6HA#4* transgenic plants were significantly higher than those of the Col-0 at 12 DAP (Figure 3). These results demonstrated that LuAccD contributes to seed FA accumulation by up-regulating the expression of *AtBCCP1*, *AtBCCP2*, *AtMCAT*, *AtKASI*, *AtKASII*, *AtSSI2*, *AtFAD2*, *AtFAD3*, and *AtPDAT2* during seed development in *A. thaliana*.

### 2.4. LuAccD Promotes Seed Germination under Salt and Mannitol Stresses in A. thaliana

**Figure 4:**
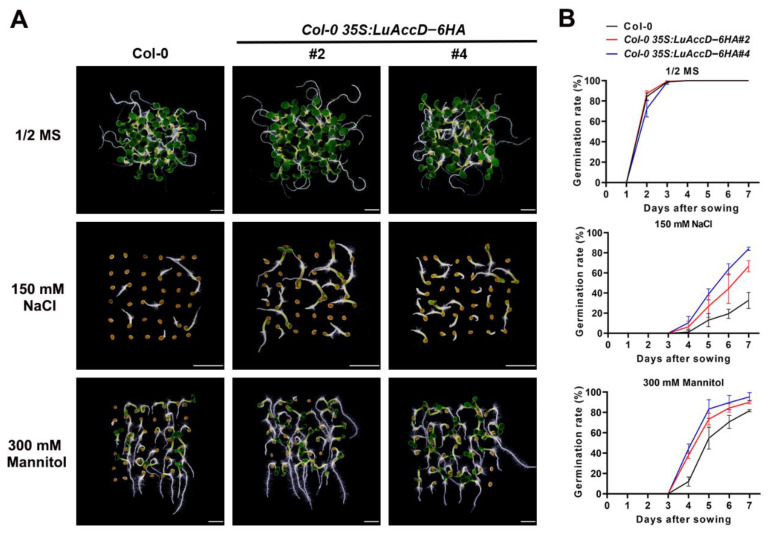
Response of wild-type (Col-0) and transgenic plants overexpressing *LuAccD* to NaCl and mannitol in seed germination. (**A**) Germination phenotype of seeds from the different lines grown on 1/2 MS plates or 1/2 MS plates with 150 mM NaCl or 300 mM mannitol for 7 days after sowing. Bar = 2 mm. (**B**) Germination rates of seeds from the different lines grown on 1/2 MS plates or 1/2 MS plates with 150 mM NaCl or 300 mM mannitol. Seed germination percentages were quantified every day from 1st day to the 7th day after sowing, and the embryonic axis protrusion was considered as seed germination. Date are the means ± SD (n = 3). Error bars denote SD. The value of each biological replicate was the average calculated over three technical replicates. For each technical replicate, we recorded the germination rates of 150 seeds from the same batch.

To determine the effects of LuAccD in response to abiotic stresses, seed germination of Col-0 and *Col-0 35S: LuAccD–6HA* plants were observed on MS agar medium containing 150 mM NaCl or 300 mM mannitol. As shown in Figure 4, Col-0 and *Col-0 35S: LuAccD–6HA* lines displayed similar germination rates and seedling growth on the medium without stress treatment (Figure 4). However, the seed germination rate of *Col-0 35S: LuAccD–6HA* lines was higher than that of Col-0 under the stress of 150 mM NaCl or 300 mM mannitol (Figure 4). Therefore, we indicated that LuAccD positively regulates the resistance of salt and mannitol stresses during seed germination in *A. thaliana*.

### 2.5. LuAccD Inhibits Expression Levels of Several Genes Contributing to ABA Biosynthesis and Signal Transduction

**Figure 5:**
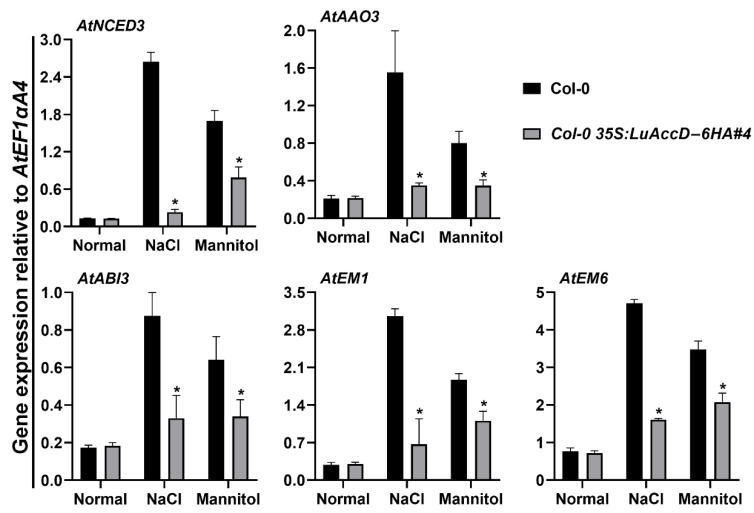
Comparison of relative transcript levels of ABA-related genes between wild-type (Col-0) and *Col-0 35S: LuAccD–6HA#4* transgenic plants. Total RNA was extracted from germinating seeds grown on the 1/2 MS or 1/2 MS containing 150 mM NaCl or 300 mM mannitol at 12 h after sowing. The expression levels of genes were calculated relative to that of the internal control *AtEF1αA4*. Values represent means ± SD (n = 3). Asterisks (*) represent significant differences between Col-0 and *Col-0 35S: LuAccD–6HA#4* lines (two-tailed paired Student’s *t*-test, *p* ≤ 0.05).

To better understand how LuAccD influences seed germination in response to salt and mannitol stresses, we assessed the expression of five ABA-related genes in Col-0 and *Col-0 35S: LuAccD–6HA#4* transgenic seeds at 12 h after sowing. As illustrated in Figure 5, there were no significant differences in the expression levels of *AtNCED3* (*NINE-CIS-EPOXYCAROTENOID DIOXYGENASE 3*), *AtAAO3* (*ABSCISIC ALDEHYDE OXIDASE 3)*, *AtABI3* (*ABSCISIC ACID INSENSITIVE 3*), *AtEM1* (*EARLY METHIONINE-LABELED 1*), and *AtEM6* between Col-0 and *Col-0 35S: LuAccD-6HA#4* transgenic seeds under the normal condition. The treatment of 150 mM NaCl or 300 mM mannitol remarkably induced the expression of these genes in both Col-0 and *Col-0 35S: LuAccD–6HA#4* germinating seeds. But the expression levels of these genes were always lower in *Col-0 35S: LuAccD–6HA#4* transgenic lines than those in Col-0 (Figure 5). These results suggested that overexpression of *LuAccD* inhibits the expression of *AtNCED3*, *AtAAO3*, *AtABI3*, *AtEM1*, and *AtEM6*, which weakens the ABA biosynthesis and ABA signal transduction, thereby resulting in the low sensitivity of transgenic plants to salt and mannitol stresses during seed germination.

## 3. Discussion

Flax is an important oil-producing crop that has attracted interest due to its high content of C18:3. Flax with improved tolerance to stresses can also be used to expand cultivation into currently undeveloped and marginal lands [33]. Therefore, it is desirable to generate elite flax germplasm with a high oil content and resistance to environmental stresses, including salt and drought stresses. In this study, we found that *LuAccD* promotes the seed FA accumulation and facilitates seed germination under salt and mannitol stresses in *A. thaliana*.

The previous study showed that *AtAccD* is essential for FA biosynthesis [32]. Consistently, we found that ectopic expression of *LuAccD* significantly promotes the accumulation of seed total FAs and major FA compositions in *A. thaliana* (Figure 2D,E). The high percent identity of carboxyltransferase domains, which play an important role in FA biosynthesis [34], was observed between LuAccD and AtAccD (Figure 1A). Therefore, we inferred that LuAccD exhibits a conserved role with AtAccD in regulating the FA accumulation of *A. thaliana* seeds. Inconsistently, the overexpression of LuAccD in Col-0 did not alter the 1000-seed weight (Figure S1). This might be ascribed to the fact that other seed components affecting seed weight, such as storage proteins, offset the higher seed total FA content in *LuAccD* transgenic seeds. The exact explanation needs to be supported by further experimental results. Notably, seed coat color, seed length and width were also not altered (Figure S1). These results indicated that *LuAccD* can be regarded as a valuable potential for flax molecular breeding.

The collaborative expression of genes participating in FA biosynthesis is important for the oil accumulation in seeds [35,36,37]. Overexpression of *LuAccD* induced the transcript levels of several genes involved in oil biosynthetic processes, including FA biosynthesis and modification, and triacylglycerol deposition, which, in turn, contributes to oil accumulation in seeds (Figure 3). Of these enzymes, *BCCP1* and *BCCP2*, like *AccD*, also encode the subunit of ACCase, which functions as a sensor or gating system that controls the overall flux of FA biosynthesis [38,39]. MCAMT converts malonyl-CoA and ACYL CARRIER PROTEIN (ACP) into CoA and malonyl-ACP, which is a key building block for the FA biosynthesis [40]. Therefore, the up-regulated expression of *AtBCCP1*, *AtBCCP2*, and *AtMACT* by LuAccD should increase the overall flux of seed FAs at the early stage of the FA biosynthetic pathway in *A. thaliana*. Additionally, three separate condensing enzymes, or 3-ketoacyl-ACP synthases (KASI–KASIII), are essential for the production of C18 FAs. Among them, KASI participates in the conversion of acetyl-ACP to palmitoyl-ACP, whereas KASII mainly utilizes palmitoyl-ACP as the substrate to produce stearoyl-ACP [41]. Studies have shown that the deficiency of KASI leads to disrupted embryo development before the globular stage and noticeably decreases seed total FA content (~33.6% of the wild-type) in *A. thaliana* [42]. SSI2 (FAB2) encodes a stearoyl-acyl carrier protein desaturase that converts C18:0 into C18:1 [43]. FAD2 catalyzes the conversion of C18:1 to C18:2 which is further desaturated by FAD3 to form C18:3 [44,45,46]. Thus, the highly up-regulated expression of *AtKASI*, *AtKASII*, *AtSSI2*, *AtFAD2*, and *AtFAD3* in *Col-0 35S: LuAccD–6HA* would accelerate the accumulation of FAs in seeds at the middle stage of the biosynthetic pathway. *PDAT2* encoding a phospholipid: diacylglycerol acyl-transferase promotes triacylglycerol production [47]. Therefore, ectopic expression of *LuAccD* in *A. thaliana* could trigger multiple transcriptional regulatory events that affect FA accumulation in seeds.

Seed germination is a critical checkpoint for crop survival under adverse conditions, and ABA plays a critical role in affecting seed germination and seedling establishment, especially under abiotic stresses [48,49,50,51]. In our study, we found that overexpression of *LuAccD* in Col-0 weakens the sensitivity of the transgenic seeds to salt and mannitol during germination (Figure 4). At the cellular level, the transcript levels of five stress-response genes, *AtNCED3*, *AtAAO3*, *AtABI3*, *AtEM1*, and *AtEM6*, were higher in Col-0 germinating seeds than in the *Col-0 35S: LuAccD–6HA* under the NaCl or mannitol stress (Figure 5). *AtNCED3* encodes 9-cis-epoxy carotenoid dioxygenase which functions in osmotic stress-induced ABA biosynthesis in *A. thaliana* [52]. It is highly induced by salt and drought stresses, and its inactivation is responsible for enhanced germination upon salt stress [53,54,55]. *AtAAO3* encodes an enzyme that catalyzes the final step of ABA biosynthesis [56], and the *Oryza sativa OsAAO3* mutation exhibited earlier seed germination [57]. AtABI3 as a major downstream component of ABA signaling has been long recognized as a master regulator of seed dormancy and ABA inhibition of seed germination [50]. The higher percentage of seed germination was observed in Atabi3 mutant compared to wild-type when exposed to ABA, mannitol or NaCl treatments [58]. *AtEM1* and *AtEM6* encoding the late embryogenesis abundant proteins are ABA-responsive marker genes, which are induced by ABA, salt and osmotic stresses [59,60,61]. Owing to these results, we concluded that the lower expression of *AtNCED3*, *AtAAO3*, *AtABI3*, *AtEM1*, and *AtEM6* caused by the overexpression of *LuAccD* in *A. thaliana* attenuates the ABA biosynthesis and ABA signal transduction, thereby resulting in low sensitivity of *A. thaliana* to salt and mannitol stresses during seed germination.

## 4. Materials and Methods

### 4.1. Plant Materials and Growth Conditions

All *A. thaliana* materials used in this study were in the Columbia ecotype (Col-0) background, and were grown in a growth chamber at 22°C with a 16/8 h light/dark cycle, which has been reported in detail previously [62].

### 4.2. Gene Cloning and Plasmid Construction

The protein sequence of AtAccD (ATCG00500) was used for protein blast against the *L. usitatissimum* reference genome (https://phytozome-next.jgi.doe.gov/pz/portal.html, accessed on 12 October 2020). One identified highly conserved sequence Lus10002473 was named *LuAccD*. The template cDNA was synthesized from total RNA extracted from germinated seeds of oil flax cultivar ‘Longya 10’. The full-length CDS of *LuAccD* without the stop codon was amplified using specific primers by PCR and was cloned into the pGreen-35S–6HA vector, forming the *35S: LuAccD–6HA* fusion vector. Primer information for the plasmid construction is given in Table S1.

### 4.3. Analysis of Protein Sequence and Phylogenetic Tree

The protein sequence of LuAccD was obtained from Phytozome (https://phytozome-next.jgi.doe.gov/pz/portal.html, accessed on 12 October 2020). Multiple sequence alignment of AtAccD and LuAccD proteins was carried out using MUSCLE website (https://www.ebi.ac.uk/Tools/msa/muscle/, accessed on 15 October 2022). The NCBI Conserved Domain Database (https://www.ncbi.nlm.nih.gov/Structure/cdd/wrpsb.cgi, accessed on 20 October 2022) was used to predicate the conserved domain of LuAccD. The phylogenetic tree was constructed by the Neighbor-Joining (NJ) method using MEGA 7.0 software with 1000 bootstrap replications and the *p*-distance model.

### 4.4. Generation of A. thaliana Transgenic Plants

The construct of *35S: LuAccD–6HA* was transformed into the *Agrobacterium tumefaciens* strain GV3101, which was then introduced into Col-0 via the floral dip method [63]. The T_1_ transgenic plants were selected by Basta^®^ (Bayer, Langenfeld, Germany) on soil and identified by using PCR in DNA level. T_2_ and T_3_ seeds were screened on 1/2 MS medium (pH 5.7, 1% sucrose, 1% agar) containing 10 μg/mL glufosinate-ammonium, and positive seedlings were transferred to soil. The T_3_ generation homozygous plants were used for subsequent experiments after cultivation under similar conditions.

### 4.5. RNA Extraction and qRT-PCR Analysis

The total RNA samples were isolated using the MiniBEST Plant RNA extraction kit (Takara Bio, Dalian, China). RNA reverse reaction was carried out with the PrimeScript RT kit (Takara Bio, Dalian, China). qRT-PCR was performed using an SYBR Green Mix (Takara Bio, Dalian, China) on a Quant Studio 7 real-time system. The relative expression values were normalized to that of the internal control *AtEF1αA4*. Statistical data were obtained from three biological replicates. For each biological replicate, two technical repetitions were performed. Primer information for qRT-PCR is given in Table S1.

### 4.6. Microscopic Observation of A. thaliana Seed Traits

The *A. thaliana* seeds were harvested from the siliques at the basal part of the major inflorescences. The mature seeds were imaged under an SZ61 stereomicroscope (Olympus, Tokyo, Japan), and their length and width were determined with ImageJ 1.48v software. The 1000-seed weight was measured by using a 0.0001 precision test analytical balance (BSA124S-CW, Sartorius, Beijing, China). Three independent biological replicates and three technical replicates were performed. For seed size measurement, each technical replicate contains 300 seeds.

### 4.7. Measurement of Seed FAs

Isolation and determination of FAs were performed according to previously described [64]. In brief, seed FAs were methylated in the 2.5% (*v*/*v*) H_2_SO_4_ solution diluted with methanol at 80 °C for 2 h. After cooling to room temperature, the solution was added with 2 mL of 0.9% (*w*/*v*) NaCl and 2 mL of hexane in due order, and the organic phase was analyzed by gas chromatography using GC-2010 plus instrument (Shimadzu, Kyoto, Japan) with a flame ionization detector and a 30 m (length) × 0.25 mm (internal diameter) × 0.5 μm (liquid membrane thickness) column (Supelco wax-10, Supelco, Shanghai, China). Methyl heptadecanoate was used as an internal standard. The initial column temperature was maintained at 160 °C for 1 min, increased by 4 °C min^−1^ to 240 °C, and held for 16 min at the final temperature. The peak for each FA composition was identified by their unique retention time, and their concentrations were calculated against the internal control.

### 4.8. Determination of Seed Germination

The *A. thaliana* seeds used for the germination analysis were harvested from plants grown under the same conditions at the same time and allowed to mature at room temperature for 3 months. The A. thaliana seeds were surface sterilized with 75% ethyl alcohol and were subsequently sown on 1/2 MS solid medium supplemented with or without 150 mM NaCl or 300 mM mannitol. The seeds were stratified at 4°C for 2 days in darkness and were then placed in the climate chamber. The germination (emergence of radicles) rate was scored daily. After 7 days, the seedlings were photographed. Seed germination percentages were quantified every day from 1st day to the 7th day after sowing, and the embryonic axis protrusion was considered as seed germination. Date are the means ± SD (n = 3). Error bars denote SD. The value of each biological replicate was the average calculated over three technical replicates. For each technical replicate, we recorded the germination rates of 150 seeds from the same batch.

## 5. Conclusions

In this study, our results demonstrated that LuAccD exhibits a conserved role with AtAccD in promoting the seed FA accumulation in *A. thaliana*. Meanwhile, LuAccD could enhance the tolerance to salt and mannitol stresses during seed germination in *A. thaliana*. In this regard, LuAccD can be utilized as a potential target for the breeding of flax varieties with high FA content and stress tolerance.

## Data Availability

All data included in this study are available upon reasonable request by contact with the corresponding author.

## Supplementary Materials

The following supporting information can be downloaded at: https://www.mdpi.com/article/10.3390/plants12173100/s1, Figure S1. Morphological observation of mature *A. thaliana* seeds randomly selected from wild-type (Col-0) and overexpression transgenic plants carrying *LuAccD* (*Col-0 35S: LuAccD–6HA#2* and *#4*). Table S1. The primers used in this study. Table S2. Comparison of percent identity between the amino acid sequences of AtAccD and LuAccD.
