# Supplementary material for: Linum usitatissimum AccD Enhances Seed Fatty Acid Accumulation and Tolerance to Environmental Stresses during Seed Germination in Arabidopsis thaliana
Source: Plants (Basel). 2023 Aug 29;12(17):3100. doi: 10.3390/plants12173100 (PMC10489840; doi:10.3390/plants12173100)
Supplement: Supplementary file 1 [file plants-12-03100-s001.zip › plants-2550004-supplementary.pdf]

Table S1. The primers used in this study.

Primer name	Primer sequences (5′-3′)	Annotations
AtEF1aA4	CTGGAGGTTTTGAGGCTGGTA<br>CCAAGGGTGAAAGCAAGAAGA	qRT-PCR
LuAccD	GAGCTTGATGCAAATGGCGAA<br>CTCTTTTACCCGCAAACGCAA	qRT-PCR
AtBCCP1	TCACTCAAACCTCCTCGCAC<br>TTGTGCTTTCACCACAGGGT	qRT-PCR
AtBCCP2	AACCCAATGGGATCTCCTTTCCCT<br>ATAAATTCAGAGAGCTCGGCGGGT	qRT-PCR
AtMCAT	GATACTTGCACGCCAGGTGA<br>CCTTTCCAGGTCCCAATTCGT	qRT-PCR
AtKASI	TCGATTTCAACTGCTTGTGC<br>CCTCCCAACCCAATAGGAAT	qRT-PCR
AtKASII	TGCCTATCACATGACCGAGC<br>CCAAAACAGTGAGCAAGGGC	qRT-PCR
AtSSI2	CGCTGTGCATAAGCATTCTC<br>TTGGGGCCGGAGCTGAGAGC	qRT-PCR
AtFAD2	ATGGGTGCAGGTGGAAGAAT<br>CCAGGAGAAGTAAGGGACGA	qRT-PCR
AtFAD3	CCACAGTACTCGGATGCTCAGA<br>GCAATAAGCTTTCTCTCGCTTGGA	qRT-PCR
AtPDAT2	AGATGATGAGACGAGCCGAAGC<br>TCTCTGGTGCCTCCGGTAATTTG	qRT-PCR

AtNCED3	GCTGCGGTTTCTGGGAGAT<br>GTCGGAGCTTTGAGAAGACGAT	qRT-PCR
AtAAO3	GGAGTCAGCGAGGTGGAAGT<br>TGCTCCTTCGGTCTGTCCTAA	qRT-PCR
AtABI3	GCGATGAAGCAGCTTTTAAAGCGAATT<br>GTCGGAGCTTTGAGAAGACGAT	qRT-PCR
AtEM1	TAGGGCACGAGGGTTATCAG<br>CGCTCTCCACCAGATTTTTC	qRT-PCR
AtEM6	GCAAACTCGAAAGGAGCAGT<br>TAGGGCACGAGGGTTATCAG	qRT-PCR
35S: LuAccD–6HA–EcoRI-F<br>*35S: LuAccD–6HA–Xmal-R*	GATAAGCTTGATATCGAATTCATGACTAGTTCAGATAGAAT<br>GTATGGGTAACTAGAACTAGTATGAGTCAAAGCGTGGAGA	*35S: LuAccD–6HA*
35S_P	GACCCTTCCTCTATATAAGGAAGTTC	Mutant genotyping

Table S2. Comparison of percent identity between the amino acid sequences of AtAccD and LuAccD.

Protein domain	Protein name	AtAccD	LuAccD
Full-length protein	AtAccD	100.0%	
	LuAccD	58.4%	100.00%
Crotonase-like superfamily domain	AtAccD	100.00%	
	LuAccD	61.5%	100.00%

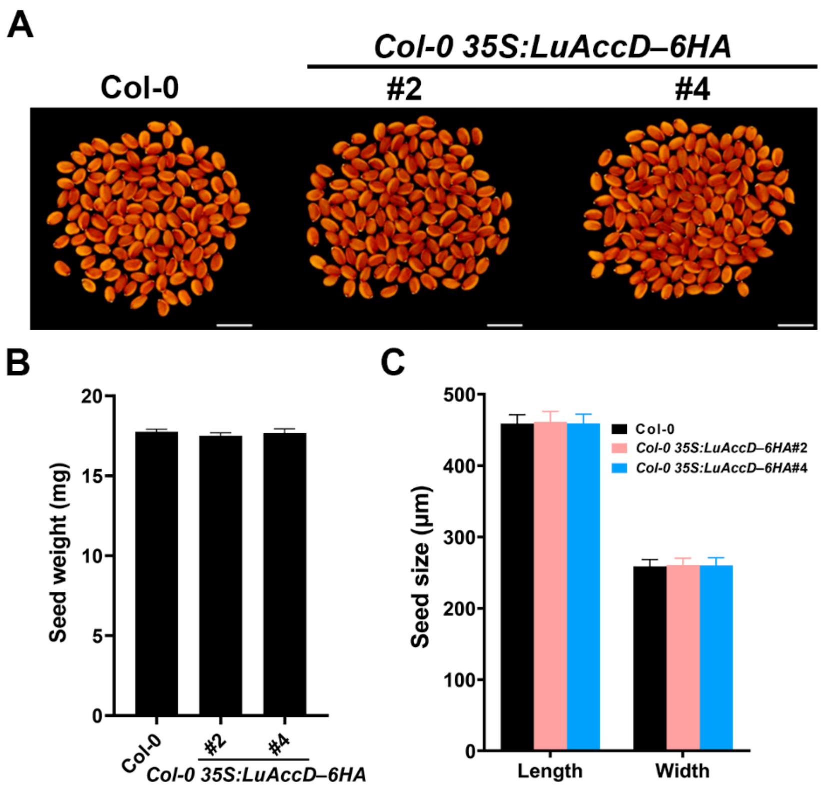

Figure S1. Morphological observation of mature *A. thaliana* seeds randomly selected from wild type (Col-0) and overexpression transgenic plants carrying *LuAccD* (*Col-0 35S: LuAccD–6HA#2 and #4*). Comparison of seed coat color (A), 1000-seed weight (B), and seed size (C) between Col-0 and *Col-0 35S: LuAccD–6HA* transgenic plants. Bar: 2 mm. There was no significant difference at $P \leq 0.05$ based on the Student's *t*-test among Col-0 and *Col-0 35S: LuAccD–6HA* transgenic plants. Seeds were harvested from 12 individual plants grown in different pots arranged randomly within one of three blocks. Values are means ± SD, and error bars denote SD. Three independent biological replicates and three technical replicates were performed for each experiment. For seed size measurement, each technical replicate contains 300 seeds.
